# In Situ Imaging for Monitoring Biomass Content and Pellet Size in Submerged Filamentous Stirred‐Tank Cultivation

**DOI:** 10.1002/biot.70314

**Published:** 2026-09-28

**Authors:** Lukas Hartmann, Tarek Wieland, Linus Witucki, Mike Barth, Dirk Holtmann

**Affiliations:** ^1^ Karlsruhe Institute of Technology Karlsruhe Germany

**Keywords:** *Aspergillus oryzae*, bioprocess analytics, fungal biomass, in situ monitoring, pellets

## Abstract

Filamentous microorganisms play a central role in industrial biotechnology, where pellet morphology affects mass transfer, rheology, and process performance. However, time‐resolved monitoring of filamentous biomass and pellet size dynamics in stirred‐tank reactors remains challenging due to offline sampling limitations. We present a noninvasive in situ imaging approach to observe biomass‐related light scattering and projected pellet size. A custom‐built camera and ring‐light setup with MATLAB‐based image acquisition and processing was used to quantify image luminosity as a proxy for biomass content and to determine projected object area (POA) and the number of detected objects per image above a POA threshold of 0.5 mm^2^. To characterize systematic biases, polyoxymethylene spheres of 1–4 mm diameter served as a model system. Luminosity scaled with object concentration, whereas object detection and apparent size estimation were increasingly affected by object size and concentration. In a bench‐scale cultivation of malic acid‐producing *Aspergillus oryzae*, image luminosity followed biomass content, while in situ POA analysis revealed increasing projected pellet size coinciding with Zn^2+^ limitation. These observations differed from ex situ sampling, indicating that image‐based in situ monitoring provides time‐resolved information on biomass formation and pellet size dynamics that may not be captured by offline sampling alone.

## Graphical Abstract and Lay Summary

Noninvasive imaging through a stirred‐tank reactor wall enabled time‐resolved monitoring of biomass and projected pellet size. Model‐sphere validation identified size‐ and concentration‐dependent biases; in *Aspergillus oryzae* cultivation, luminosity followed biomass, pellet‐size increases coincided with Zn^2+^ limitation, and in situ trends differed from ex situ measurements.

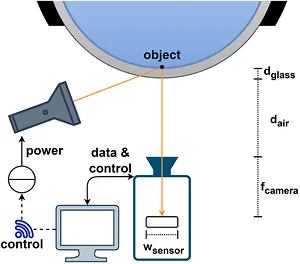

## 1 Introduction

Filamentous microorganisms play a central role in industrial biotechnology, with *Aspergillus niger* being a prominent example due to its widespread use in the large‐scale production of citric acid [1]. In research, filamentous bacteria such as *Streptomyces* spp. are also exploited for the production of antibiotics and other bioactive compounds [2]. These organisms grow as branching hyphae, potentially aggregating into dense structures known as pellets. The morphology of such pellets has a direct impact on mass transfer, rheology, and overall process performance [3, 4, 5]. Consequently, monitoring both biomass growth and morphology is essential for process optimization.

Growth monitoring of filamentous cultures is inherently more challenging than in unicellular cultivations. Methods commonly used for monitoring growth of bacteria and yeast, such as optical density (OD) measurements, whether manual or using inline probes, are often unreliable in the application to filamentous organisms due to their variable macromorphology, ranging from dispersed hyphae and fragments to clumps and pellets. Dry weight determination is a widely used offline method for biomass quantification that involves filtration or centrifugation, washing, drying, and weighing of the biomass. While it provides absolute values, it is time‐consuming, destructive, and offers only low temporal resolution, making it unsuitable for dynamic monitoring or high‐throughput applications. Packed mycelial volume estimates biomass content by measuring the volume occupied by sedimented biomass [6]. This method is simple and fast, but suffers from the porous and irregular mycelial structure, trapping liquid and air inside the pellet mass. Methods relying on manual sampling and processing are inherently prone to variability and can compromise data accuracy. Online monitoring options include dielectric spectroscopy using capacitance probes, estimating viable biomass from the dielectric response of intact cellular membranes [7, 8]. Although effective, these probes require integration into the bioreactor system, which may limit their suitability for academic laboratories or parallelized small‐scale setups. Alternatively, soft sensors based on indirect measurements, such as fluorescence signals, off‐gas stream composition, substrate consumption rates, or dissolved oxygen (DOT), can be used to infer biomass growth in real time [9, 10, 11]. These approaches rely on mathematical models or empirical correlations and offer noninvasive, scalable solutions for dynamic monitoring, although they require careful calibration and validation to ensure reliability.

Besides biomass formation, morphology is a key determinant of process performance of filamentous organisms; however, monitoring morphology in a quantitative and time‐resolved manner remains difficult. Offline methods, such as stereomicroscopy or light microscopy combined with manual or automated image analysis, are commonly used to assess pellet size, shape, and structure, including parameters such as area, Feret's diameter, aspect ratio, and solidity [12, 13, 14]. While these approaches provide detailed morphological descriptors, they are labor‐intensive, destructive, and limited to discrete time points, requiring manual sampling and object fixation. Flow cytometry has also been applied to analyze morphological features such as hyphal length, enabling high‐throughput, in‐flow analysis of large particles with acquisition of scatter signals, fluorescence profiles, and in‐flow images, but it requires sample preparation, including dilution and staining [15]. Online methods are less established and typically rely on advanced instrumentation such as in situ microscopy or focused beam reflectance measurement [16, 17]. Additional imaging approaches include bright‐ and dark‐field probes for online visualization, as well as phase‐contrast and epi‐illumination microscopy for imaging cells and microcarriers [18, 19, 20, 21]. Digital holographic imaging offers a label‐free and noninvasive option for quantitative cell and morphology analysis, although its implementation in dense, moving, and optically complex pellet cultures remains technically demanding [22].

Existing approaches are well suited for microscopic samples or defined optical configurations, but their direct application to real‐time, non‐destructive monitoring of millimeter‐scale fungal pellets in stirred‐tank reactors (STR) remains challenging. To address this gap, we developed an in situ imaging pipeline that combines biomass estimation via light scattering, inferring biomass content from changes in overall image luminosity, with automated analysis of projected pellet size based on object detection using MATLAB algorithms. The pipeline relies on readily available components including a standard industrial camera, a ring light, and a support structure assembled using common laboratory scaffolding materials, facilitating accessibility and potential scalability, as shown in Figure 1A. To systematically evaluate potential biases and technical constraints, a controlled model setup employing polyoxymethylene (POM) spheres was implemented to mimic pellet‐like structures. Subsequently, the monitoring system was applied to a bench‐scale cultivation of *Aspergillus oryzae*, a filamentous fungus investigated for malic acid production [23]. Complementary process data from off‐gas analysis, metabolite profiling, and offline biomass characterization were integrated to contextualize image‐based observations.

**FIGURE 1:**
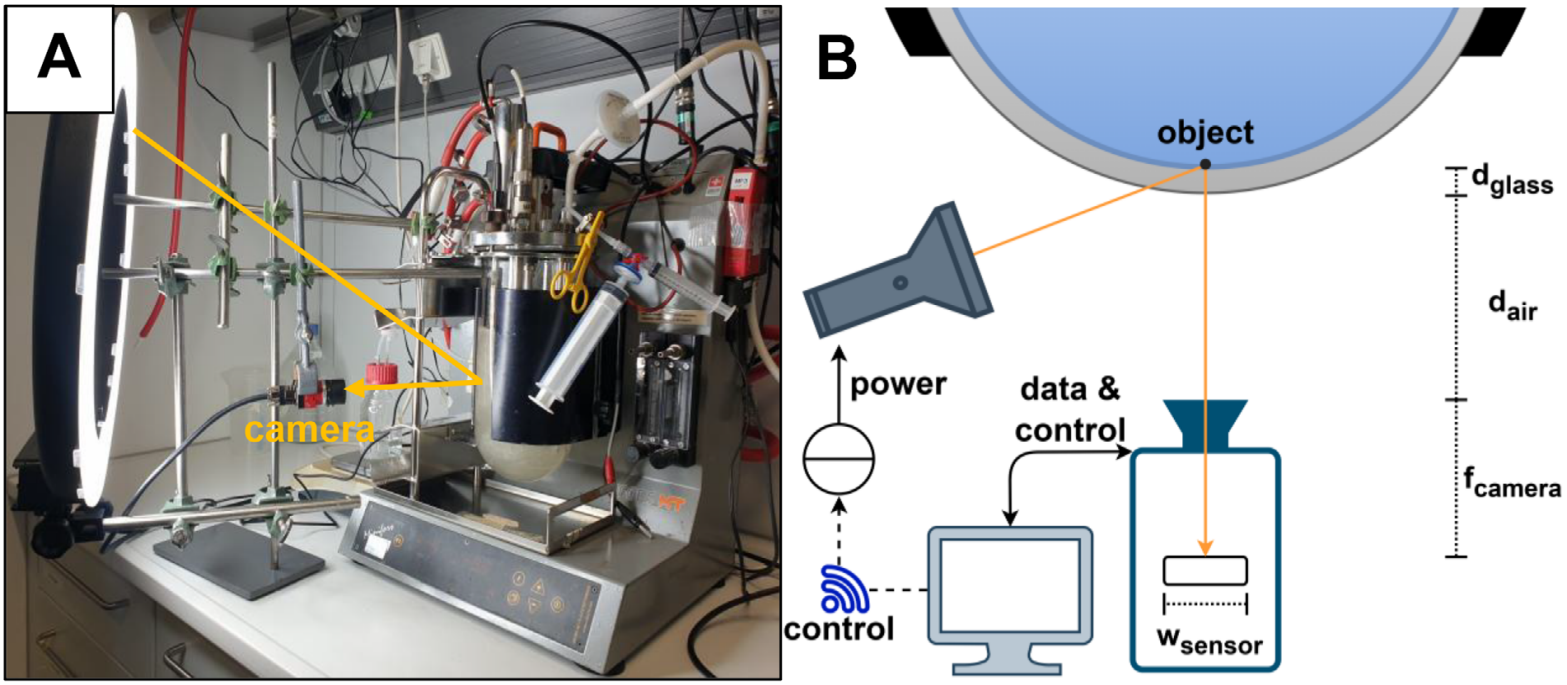
Optical setup in laboratory (A) and illumination geometry (B). The spatial resolution *r* in Equation (1) was calculated with the defined geometric distances of *d*_air_ = 173 mm and *d*_glass_ = 5 mm. The focal length of the lens *f*_camera_ = 12 mm, and the physical width of the camera sensor *w*_sensor_ = 5.02 mm, corresponding to 1456 pixels along the horizontal axis, were obtained from manufacturer specifications. Calculations were based on the shortest distance along the direct optical axis. The spatial calibration therefore represents an approximation based on the defined imaging geometry.

## 2 Methods

This section outlines the experimental and analytical procedures used in this study. It describes the microbial cultivation, the design of the model system, and the image acquisition setup. The applied image analysis methods are then presented, followed by the normalization of luminosity to enable evaluation of the luminosity‐concentration correlation.

### 2.1 Strain Cultivation and Analytical Procedures

Aspects of cultivation strategy, medium composition, reactor setup, analytical workflows, and data processing adhered to the framework established by Hartmann et al., with the exception of the image‐based monitoring system implemented in the present study [24]. Deviations from the original protocol are explicitly noted. In short, *A. oryzae* DSM 1863 was sourced from the DSMZ strain collection (Deutsche Sammlung von Mikroorganismen und Zellkulturen GmbH, Braunschweig, Germany). Conidia propagation was performed on an agar‐based minimal medium containing glucose, NaNO_3_, KCl, and Hutner's Trace Elements [25, 26, 27, 28]. Precultures were inoculated with conidia and cultivated in shake flasks using a glucose‐ and ammonium‐based medium. Washed biomass from the preculture was subsequently used to inoculate the main culture for organic acid production in STR, which likewise relied on glucose and ammonium as carbon and nitrogen sources. Since hypoxia has been reported to promote loose mycelial growth, the air supply was increased to favor compact spherical pellets suitable for pellet‐size monitoring [29]. This adjustment, from 0.7 L_n_ min^−1^ air to 1.4 L_n_ min^−1^, represents the only deviation from the referenced protocol. Analytical procedures, including biomass and metabolite quantification, were conducted as previously described. The referenced study additionally comprises calculations of oxygen transfer rate (OTR), carbon dioxide transfer rate (CTR), respiratory quotient (RQ), and mass balances of quantified metabolites. In contrast to the referenced study, cell dry weight (CDW) is reported as concentrations in the reactor rather than values derived from a mass balance accounting for volume changes and sampling losses, in order to maintain comparability with luminosity‐based assessments [24].

### 2.2 Model System

A defined quantity of POM spheres (RGPBALL, Cinisello Balsamo, Italy) with diameters of 1, 2, 3, and 4 mm was gravimetrically counted and introduced into a reactor setup similar to the design used for cultivation experiments [24]. The reactor was filled with 1.4 L of demineralized water and operated at room temperature and an agitation frequency of 400 rpm. Installation of the sparger, sampling tube, pH probe, and DOT probe was omitted to prevent POM spheres from becoming trapped in narrow gaps and to avoid mechanical damage. In contrast to the standard cultivation setup equipped with two Rushton turbines, the lower impeller in this model system was replaced by a propeller to ensure sufficient suspension of POM spheres in the liquid phase. Image acquisition was performed in the region between the upper and lower impellers.

### 2.3 Imaging Setup

The MATLAB source codes used in this study are provided in the Supporting Information.

The camera (Alvium 1800U‐158M‐CH‐C, Allied Vision Technologies GmbH, Stadtroda, Germany), equipped with a lens (M117FM12‐RG, Tamron Co., Ltd., Saitama, Japan), was mounted on a self‐constructed stand that allowed precise adjustment of the distance between the camera and the reactor, as shown in Figure 1A. The focus point of the camera was set to the outer surface of the reactor glass facing the camera and mechanically fixed using the lens adjustment screws to maintain a constant focal setting throughout the experiments. For spatial calibration, a geometric reference distance of 178 mm, comprising an air gap of *d*_air_ = 173 mm and a glass thickness of *d*_glass_ = 5 mm, was used to determine the spatial resolution, enabling conversion of pixel dimensions into real‐world units according to Equation (1). Due to the optical configuration, objects located at different distances from the focal plane were detected in the images. Consequently, the observation depth was not strictly defined, and analysis was based on the recorded 2D projections. A ring light (BR‐RL18, Bresser GmbH, Rhede, Germany) was mounted coaxially around the camera to provide uniform illumination along the optical axis. Image acquisition during cultivation was controlled via a custom MATLAB script (R2023b, The MathWorks, Natick, Massachusetts, USA), in which a repeating scheduler script, referred to as Code 1, executed a dedicated acquisition routine, referred to as Code 2, at fixed intervals of 30 min. At each image acquisition event (IAE), the acquisition routine wirelessly activated a smart power outlet (Shelly Plug S, Shelly Group SE, Sofia, Bulgaria), supplying power to the ring light and enabling illumination. An IAE consisted of a 1 s pause followed by acquisition of a single image with an exposure time of 1014 µs. Each image was then checked to ensure complete transmission from the camera to the host computer, and only fully transmitted images were stored. This process was repeated until 30 valid images had been collected. Upon completion of the IAE, the outlet was signaled to deactivate, switching off the light source. Illumination was strictly limited to the duration of image capture. This approach was based on literature showing that light exposure can affect mycelial growth and conidium production in *A. oryzae* [30]. Ambient room lighting was minimized during IAEs to reduce background interference, except during reactor handling procedures, such as taking samples for reference measurements and metabolite quantification. For the model system, images were acquired manually using the Vimba X SDK software (Version 1.4.0.8c3b77a, Allied Vision Technologies GmbH, Stadtroda, Germany).

(1) $$r=\frac{d_{\mathrm{glass}}+d_{\mathrm{air}}}{f_{\mathrm{camera}}}\;\frac{w_{\mathrm{sensor}}}{N_{\mathrm{pixel}}}.$$

### 2.4 Image Analysis

The image analysis was conducted in a structured, multi‐step process illustrated in Figure 2 and implemented in a custom MATLAB script, referred to as Code 3. Initially, all images were converted to 8‐bit grayscale format, standardizing pixel intensity values. Subsequently, the cylindrical reactor view was geometrically rectified using camera calibration parameters obtained from a checkerboard calibration pattern positioned at the inner surface of the reactor wall. Local contrast enhancement was applied using contrast limited adaptive histogram equalization (CLAHE), improving the visibility of pellet contours and internal structures. Regions of interest (ROIs) were pre‐defined to focus the analysis on representative areas of the reactor broth, deliberately excluding zones affected by light reflections or mycelial attachments. For analysis of 2D projected object area (POA), binarization was performed using adaptive thresholding to distinguish biomass structures from the background. Agglomerate splitting was performed using watershed segmentation. Subsequently, object filtering was applied to exclude artifacts and nonrepresentative structures. This filtering was based on three criteria: a minimum POA of 0.5 mm^2^ to remove small fragments and noise; shape descriptors such as circularity ≥ 0.5 and aspect ratio ≤ 2 to avoid measuring artifacts; and a multi‐level luminosity filter. The latter included the average object luminosity, excluding overly illuminated regions such as the central light halo; the standard deviation of object luminosity, removing low‐contrast artifacts lacking internal texture; and the luminosity gradient, enabling the exclusion of air bubbles characterized by sharp edge transitions. For all remaining objects, POA was evaluated to quantify pellet size distribution, while object abundance was defined as the number of detected objects per image within the analyzed ROIs. For each IAE, object abundance was summarized as the mean and standard deviation across the corresponding images. For POM spheres, final size measurement was expressed as diameter rather than POA, as their nearly circular projections allow POA to be converted into an equivalent circular diameter. For circular projections, the validated POM sphere diameter range of 1–4 mm corresponds to a POA range of 0.8–12.6 mm^2^; objects between the applied lower POA threshold of 0.5 and 0.8 mm^2^ were not separately validated. Fungal pellets exhibit pronounced morphological heterogeneity and irregular contours; therefore, their size is represented by POA. POA values were summarized using the median and interquartile range (IQR) between the 25th and 75th percentiles. The median was selected as a measure of central tendency for size distribution and to reduce the influence of occasional measurement artifacts, as shown in Figure 2.

**FIGURE 2:**
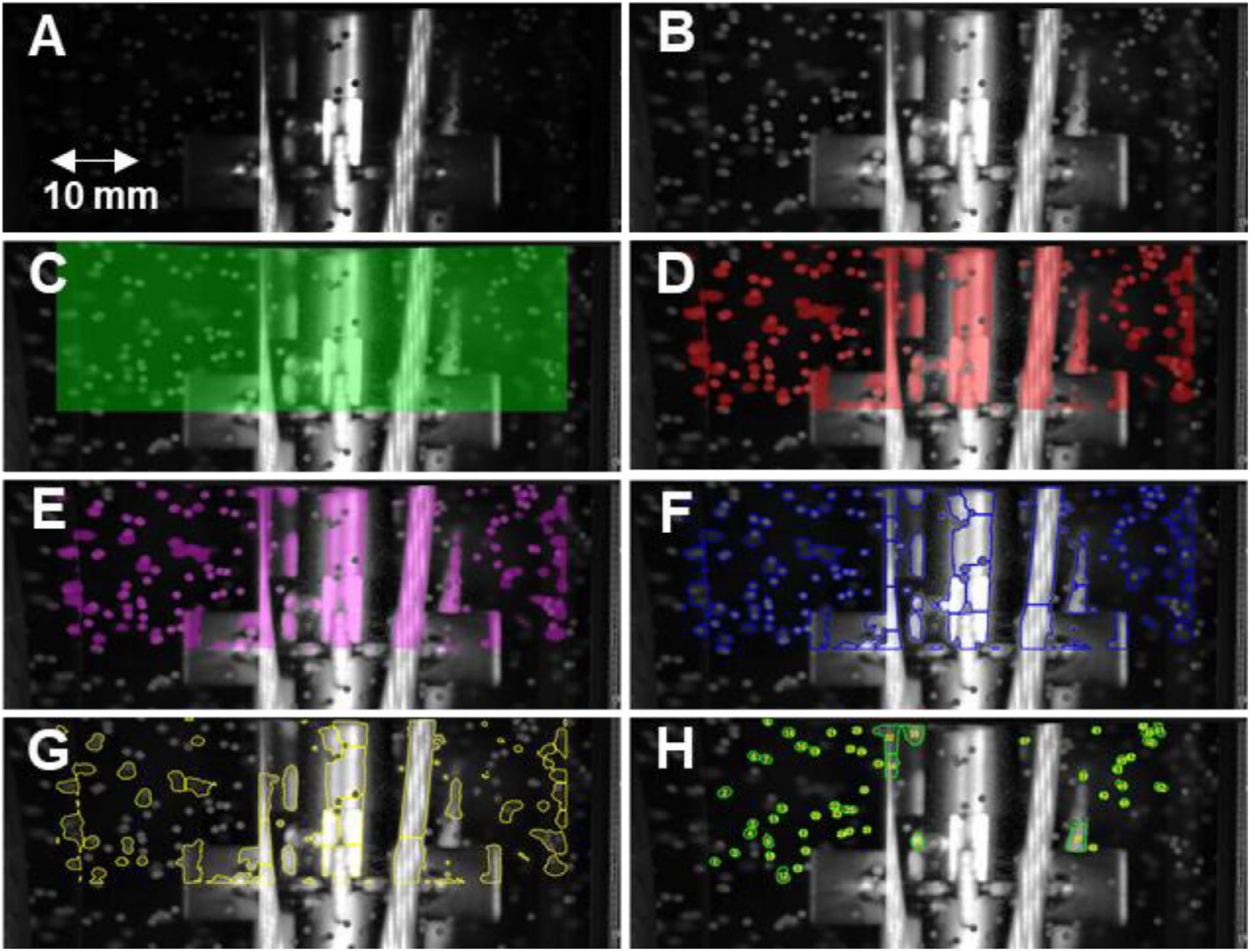
Processing logic of the image analysis pipeline illustrated by control visualizations of POM spheres as a model system in 2.5 L STR. The experiment was performed in 1.4 L of demineralized water with 3000 POM spheres of 1 mm diameter at 400 rpm without aeration. The images were created using a custom MATLAB script, referred to as Code 4. (A) Shows the original image after rectification, and (B) the contrast‐enhanced version. (C) Displays the ROI in green. (D) Highlights segmented foreground areas after binarization in red. (E) Presents the binary structures after watershed‐based segmentation in pink. (F) Shows the outline definition in blue. (G) Marks excluded regions not meeting the filter criteria in yellow. (H) Displays accepted objects outlined in green, with their assigned object IDs in yellow.

Scattered light intensity for estimation of biomass content was determined using Code 5 by calculating the mean pixel luminosity within the ROIs for each image, using the same preprocessing as for the POA analysis but without contrast enhancement, as shown in Figure 3. The resulting mean luminosity values per image were subsequently averaged across all images of each IAE. For visualization in Figure 7, the minimum value of the resulting luminosity time series was subtracted from every value in the series.

**FIGURE 3:**
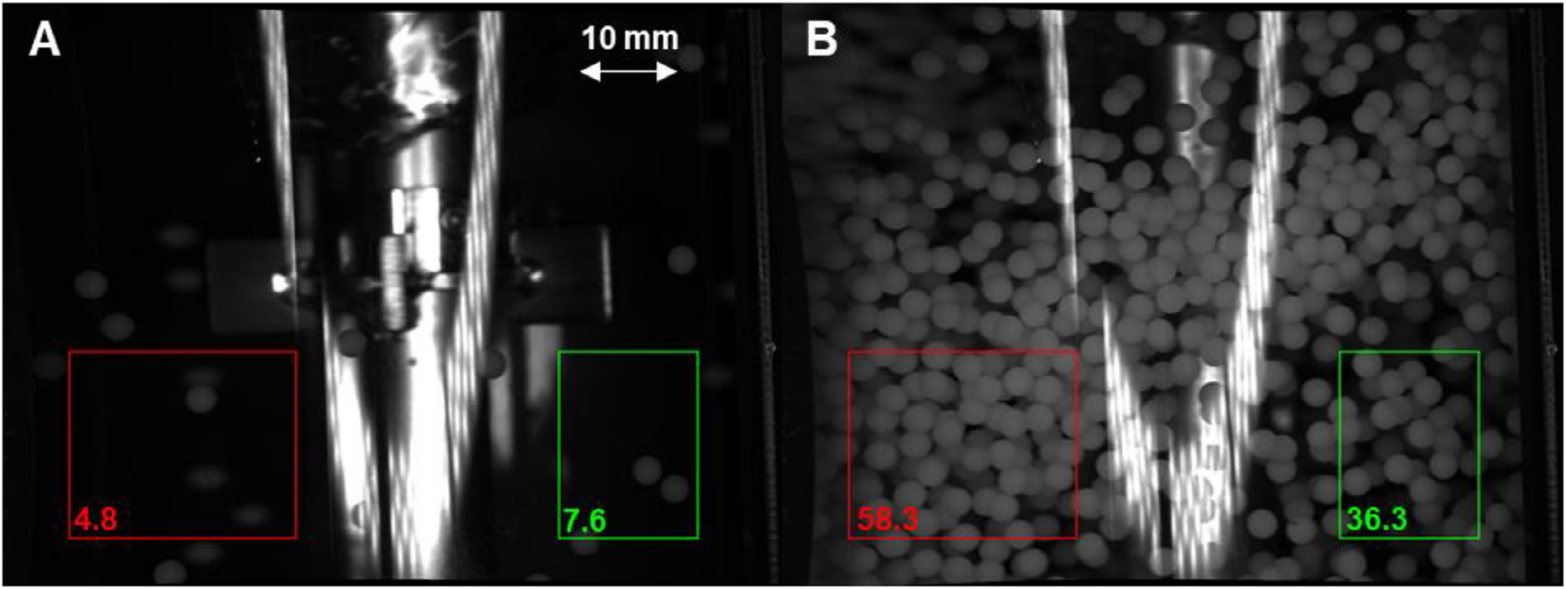
Luminosity analysis illustrated with a control visualization of POM spheres as a model system in 2.5 L STR. The images were created using a custom MATLAB script, referred to as Code 6. Spheres of 3 mm diameter were suspended in 1.4 L of demineralized water with (A) 200 POM spheres and (B) 5000 POM spheres at 400 rpm without aeration. ROIs are delineated by red and green squares. The corresponding average pixel luminosity is depicted inside the squares. Intensity of pixel luminosity ranges from 0 to 255.

The described analysis configuration for object filtering parameters and luminosity was applied to the POM sphere model system as well as to the fungal cultivation, differing only in the location of the ROI analysis to exclude areas occupied by mycelial attachments during cultivation, as well as in the luminosity parameters for object filtering to account for differences in light scattering properties between POM spheres and fungal pellets. For object filtering, POA analysis of fungal pellets applied a luminosity standard deviation ≤ 15 and an average luminosity ≤ 100 within valid objects, whereas POM sphere analysis applied values of ≤ 60 and ≤ 160, respectively.

### 2.5 Normalization of Luminosity for Luminosity‐Concentration Correlation

To relate scattered light intensity to object concentration, luminosity values were normalized separately for each sphere diameter using min–max scaling, allowing comparison of relative luminosity changes across different sphere diameters and concentrations, as shown in Figure 5A. First, the average luminosity ${\bar{L}}_{k,i}$ was calculated for each condition by averaging the luminosity values *L*_k,i, j_ of all images acquired *j* for a given sphere diameter *k* and sphere count *i*, as defined in Equation (2).

(2) $${\bar{L}}_{k,i}=\frac{1}{n_{k,i}}\sum_{j=1}^{n_{k,i}}L_{k,i,j}.$$

Next, for each sphere diameter, the minimum average luminosity across all sphere amounts was subtracted from the corresponding values of ${\bar{L}}_{k,i}$ to remove the baseline offset, resulting in offset‐corrected luminosities $L_{k,i}^{\prime }$, as defined in Equation (3). Offset correction was necessary because different sphere diameters produced different baseline luminosity levels, which would otherwise prevent direct comparison of relative luminosity changes across diameters.

(3) $$L_{k,i}^{\prime }={\bar{L}}_{k,i}-\mathrm{mi}n_{i}\left({\bar{L}}_{k,i}\right).$$

Finally, for each sphere diameter, the offset‐corrected luminosity values $L_{k,i}^{\prime }$ were divided by the maximum value observed within the same diameter group, resulting in normalized luminosities $L_{k,i}^{\prime \prime }$ ranging from 0 to 1, as defined in Equation (4).

(4) $$\;L_{k,i}^{\prime \prime }=\frac{L_{k,i}^{\prime }}{\mathrm{ma}x_{i}\left(L_{k,i}^{\prime }\right)}.$$

### 2.6 Ex Situ Sampling and Image Analysis

Conventional ex situ sampling was performed in parallel with the in situ camera measurements to provide an independent CDW reference for biomass quantification and complementary offline measurements of pellet size. 10 mL samples from the cultivation broth were filtered through a 0.5 mm stainless steel mesh to separate solid biomass. Particles passing the mesh would, assuming an approximately circular projection, have a POA below 0.2 mm^2^ and were therefore below the applied analysis threshold of 0.5 mm^2^ and not considered in the size analysis. The biomass was rinsed with demineralized water. The retained material was transferred to a Petri dish and scanned at 2400 dpi using a flatbed scanner (Perfection V600 Photo, Epson Deutschland GmbH, Düsseldorf, Germany). Subsequently, the biomass was dried at 80°C for a minimum of two days prior to gravimetric determination of CDW. CDW was reported as raw broth measurements to facilitate comparison with pixel luminosity.

The 2D scans were analyzed with custom MATLAB code for POA evaluation. This analysis provided complementary offline pellet size measurements for comparison with those obtained from in situ reactor imaging. In contrast to reactor images, the scans represented planar images acquired under uniform and controlled conditions, showing dark pellets on a bright and homogeneous background without optical distortions, reflections from curved surfaces, gas bubbles, or depth‐dependent effects. Due to these fundamentally different imaging characteristics, the image processing workflow differed from that used for reactor images.

Images were downscaled and converted to grayscale, and the Petri dish region was automatically identified using circle detection, as shown in Figure 4. A slightly smaller inner safety perimeter was defined to exclude edge artifacts and reflections, and pixels outside this region were masked. Pellets were segmented using adaptive thresholding to detect dark objects on the bright background, followed by morphological cleanup to remove small artifacts and refine object boundaries. Object properties including area, centroid, bounding box dimensions, and grayscale intensity were extracted for each segmented object, and the POA was calculated by converting pixel area into physical units using a fixed spatial calibration. Objects were retained only if they met predefined criteria including a minimum POA of 0.5 mm^2^, a maximum aspect ratio, a maximum object length, and a mean grayscale intensity threshold.

**FIGURE 4:**
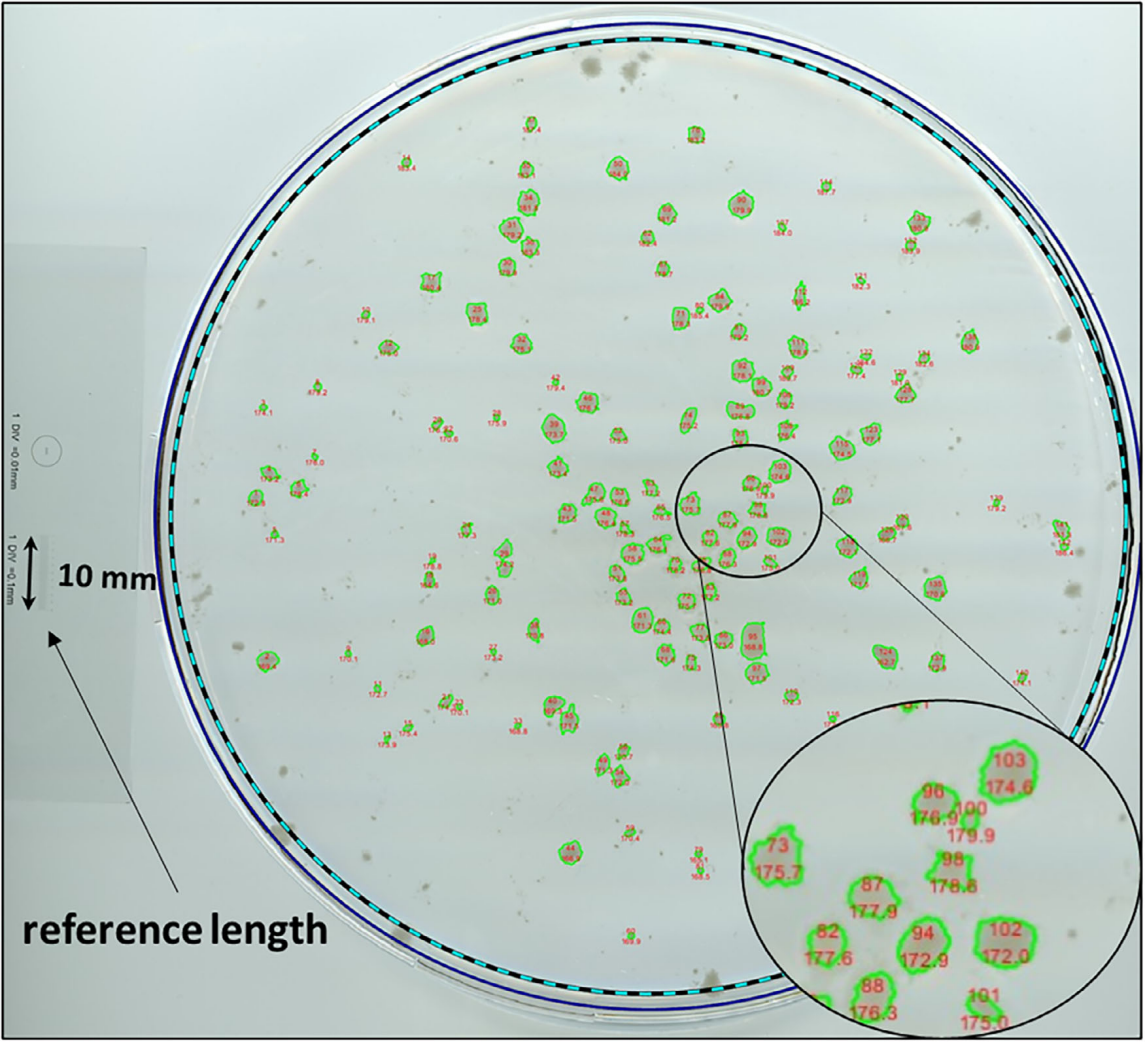
Flatbed scan of *A. oryzae* DSM 1863 pellets in a Petri dish after 19 h of cultivation, providing complementary ex situ object size measurements. The analysis was performed using a custom MATLAB script, referred to as Code 7. The Petri dish boundary is identified and marked with a blue circle, while the inner dashed circle defines a buffer zone excluding edge artifacts from analysis. Individual objects are automatically detected and outlined in green. For each detected object, the upper red number indicates the assigned object ID and the lower red number the corresponding grayscale intensity. A 10 mm scale bar is included for spatial calibration.

## 3 Results and Discussion

The performance of the in situ image analysis was first evaluated in terms of accuracy and precision using POM spheres as a defined model system. Subsequently, the method was applied to an *A. oryzae* cultivation to assess its suitability for pellet characterization during cultivation.

### 3.1 Validation of In Situ Analysis in Model System

Defined quantities of POM spheres of varying diameters were introduced into a STR setup described above. Figure 5A demonstrates that min–max normalized luminosity exhibited a strong linear correlation with the number of spheres in the reactor, showing that luminosity across the reactor's ROI scales proportionally with the number of added spheres within the tested range. In Figure 5B, the scaling relationship between sphere count and detected objects per image is shown, with power‐law fits applied to quantify deviations from proportional scaling. The exponent *b* describes the scaling behavior, with *b* < 1 indicating sublinear scaling. The smaller *b* values reflect stronger crowding effects for larger spheres and are most likely caused by overlapping and occlusion. Figure 5C shows that the precision of the estimated diameters, reflected by the width of the IQR, increased with sphere number and stabilized at higher object counts. In contrast, accuracy decreased at high sphere concentrations for larger spheres, as indicated by an increasing deviation from the nominal sphere diameter, while remaining largely stable for smaller spheres. Collectively, these findings demonstrate that image‐derived luminosity reliably reflects object content within the tested range, while image‐derived size metrics can be affected by size‐dependent crowding effects, leading to systematic deviations at higher object densities.

**FIGURE 5:**
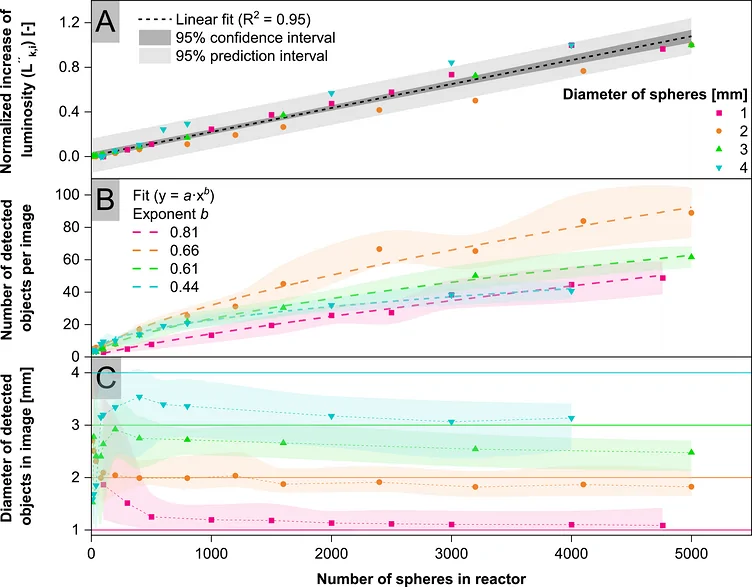
In situ characterization of POM spheres as a model system in 2.5 L STR. Incubation was performed in 1.4 L demineralized water at 400 rpm without aeration. Dashed lines in (C) and Akima‐spline‐connected deviations in (B) and (C) are provided for visual guidance. The diameter of detected objects represents the median calculated across images of an individual IAE; shaded areas represent IQRs. The colored horizontal lines in (C) indicate the nominal diameters of the spheres. The number of detected objects corresponds to the average across images. Shaded areas represent the standard deviation across images. For each condition, 10 to 20 images were analyzed. Maximum solid volume fractions were 0.18%, 1.47%, 4.81%, and 8.74% for 1, 2, 3, and 4 mm spheres, respectively, and were calculated as total sphere volume divided by total suspension volume comprising 1.4 L water and sphere volume.

To further assess potential bias in diameter estimation under heterogeneous conditions, mixtures of POM spheres with two distinct sizes were analyzed in the same STR setup. This experiment aimed to determine whether the image analysis pipeline reflects changes in size distributions when objects of different diameters coexist, as such scenarios resemble real fungal cultures where pellets vary in size.

The distributions, as shown in Figure 6, revealed that the majority of detected objects had diameters around 1 mm, which aligned with the large share of 1 mm spheres present in all mixtures. As the proportion of 2 mm spheres increased, a second cluster of detected objects appeared near 2 mm, and the median diameter shifted upward accordingly, indicating that the algorithm captured the expected trend in size composition and reflected changes in mixture ratios. However, the distributions also included objects with intermediate diameters between 1 and 2 mm, as well as values exceeding 2 mm, most likely resulting from overlapping spheres or segmentation artifacts that affected diameter estimation. Overall, the method reliably captured dominant size classes and compositional trends, although image‐derived size metrics should be interpreted with awareness of such deviations.

**FIGURE 6:**
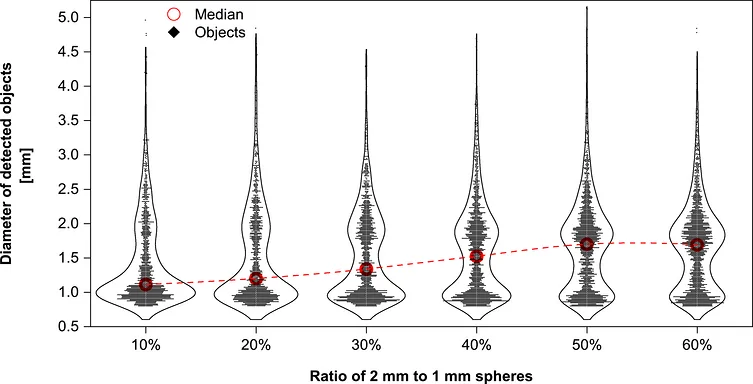
In situ characterization of POM sphere mixtures in 2.5 L STR. Incubation was performed in 1.4 L demineralized water at 400 rpm without aeration. Mixtures contain 500–3000 spheres of 2 mm diameter combined with 5000 spheres of 1 mm diameter, corresponding to the indicated ratios. For each condition, the objects depicted originate from 20 images. The numbers of detected objects were *n* = 1262, 1207, 1428, 1469, 1607, and 1678 for the 10%, 20%, 30%, 40%, 50%, and 60% mixtures, respectively.

### 3.2 In Situ Growth Analysis of *A. oryzae* Cultivation

After assessing analysis performance using defined POM spheres, the image analysis approach was applied to an *A. oryzae* cultivation to assess pellet size dynamics and object abundance under cultivation conditions. *Aspergillus oryzae* was cultivated in a 2.5 L STR under normoxic conditions by increasing the aeration rate to 1.4 L_n_ min^−^^1^, twice that of the reference process [24]. As shown in Figure 7, the increased aeration rate maintained DOT consistently above 36%, consistent with the formation of compact pellets instead of loose filaments. Glucose concentration decreased gradually but remained above 50 g L^−1^ until the end of cultivation, indicating carbon sufficiency.

**FIGURE 7:**
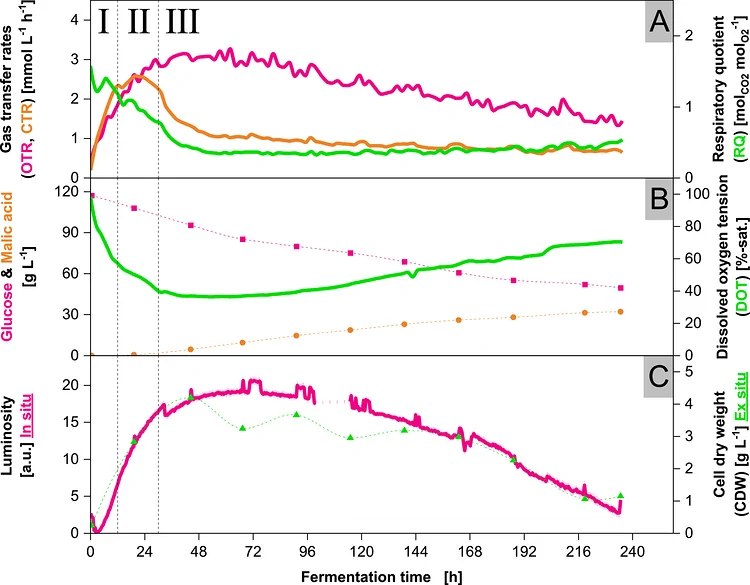
Growth monitoring of *A. oryzae* DSM 1863 in 2.5 L STR. Cultivation was performed with *X*_0_ of 7.5 g L^−1^ biomass, 109 g L^−1^ glucose, *V*_0_ of 1.4 L, 400 rpm, 32°C, and 1.4 L_n_ min^−1^ air. pH was maintained with 4 M NaOH and 4 M H_3_PO_4_ at 6.50 ± 0.05. Data for OTR, CTR, RQ, and DOT were averaged to obtain 100 data points and represent a single cultivation. Phases I, II, and III are demarcated by dashed lines based on the CTR trajectory. In situ imaging was unavailable between 101 and 115 h due to a temporary technical issue. Luminosity in situ represents the average calculated across 30 individual images for each IAE. The shaded area of luminosity corresponds to the standard deviation across the 30 images for each IAE. Dashed lines of metabolites and CDW are provided for visual guidance. Process data represent a single cultivation.

The process was divided into three phases based on characteristic changes in the CTR, reflecting metabolic transitions previously described for this strain and medium composition [24, 29]. Phase I corresponds to nutrient‐unlimited biomass growth, phase II begins with the CTR plateau associated with Zn^2+^ depletion, and phase III starts with the CTR decline indicating ammonium depletion. The RQ decreased from 1 in phase I to 0.5 in phase III, consistent with a metabolic shift toward organic acid production.

CDW was determined as an ex situ reference for absolute biomass quantification, since gravimetric measurement is well established for filamentous fungi. CDW increased steadily during phase I, reached its maximum near the CTR plateau in phase II, and declined slightly toward the end of phase III, coinciding with nitrogen limitation [24]. Image‐derived luminosity, which represents scattered light intensity across the reactor projection area, showed a similar trend. Luminosity rose sharply during early growth, peaked around the same time as CDW, and decreased gradually thereafter. This parallel behavior indicates that luminosity can serve as a proxy for biomass content, thereby providing an indirect indicator of relative biomass changes rather than an absolute measurement of biomass concentration, consistent with the proportionality observed in the model system.

A discrepancy between CDW and luminosity was observed in the middle of the cultivation period, where CDW appeared to increase between 67 and 91 h despite decreasing respiration activity, which did not indicate substantial continued biomass growth. This deviation likely reflects systematic inaccuracies inherent to the offline sampling procedure rather than true biological changes. Sampling of macroscopic 3D structures such as fungal pellets through a steel sampling port, silicone tubing, a three‐way valve, and syringe connectors potentially alters the biomass content of the collected sample. Pellet fragments may be retained or dislodged within the sampling path, occasionally leading to partial blockage during sampling and introducing variability in CDW measurements. Small peaks in luminosity coincided with manual sampling events and are likely attributed to changes in ambient lighting when the room illumination was switched on or off, resulting in the initial drop immediately after inoculation and the brief increase in luminosity shortly before process termination.

Throughout the cultivation, the control visualizations illustrated in Figure 8 reflected the biomass growth trend shown in Figure 7. At intermediate stages, the reactor showed a dense pellet population with little free space, whereas at late stages, substantially more open volume was visible between pellets, mirroring the decreasing CDW and luminosity. Furthermore, the lower section of the reactor revealed the onset of mycelial attachment from 44 h onward. We hypothesize that the decline in suspended biomass is partly attributable to the entrapment of fine fungal fragments within this attached mycelium. In addition, fungal biomass likely suffers from pellet disintegration, fragmentation, and autolysis during nitrogen starvation.

**FIGURE 8:**
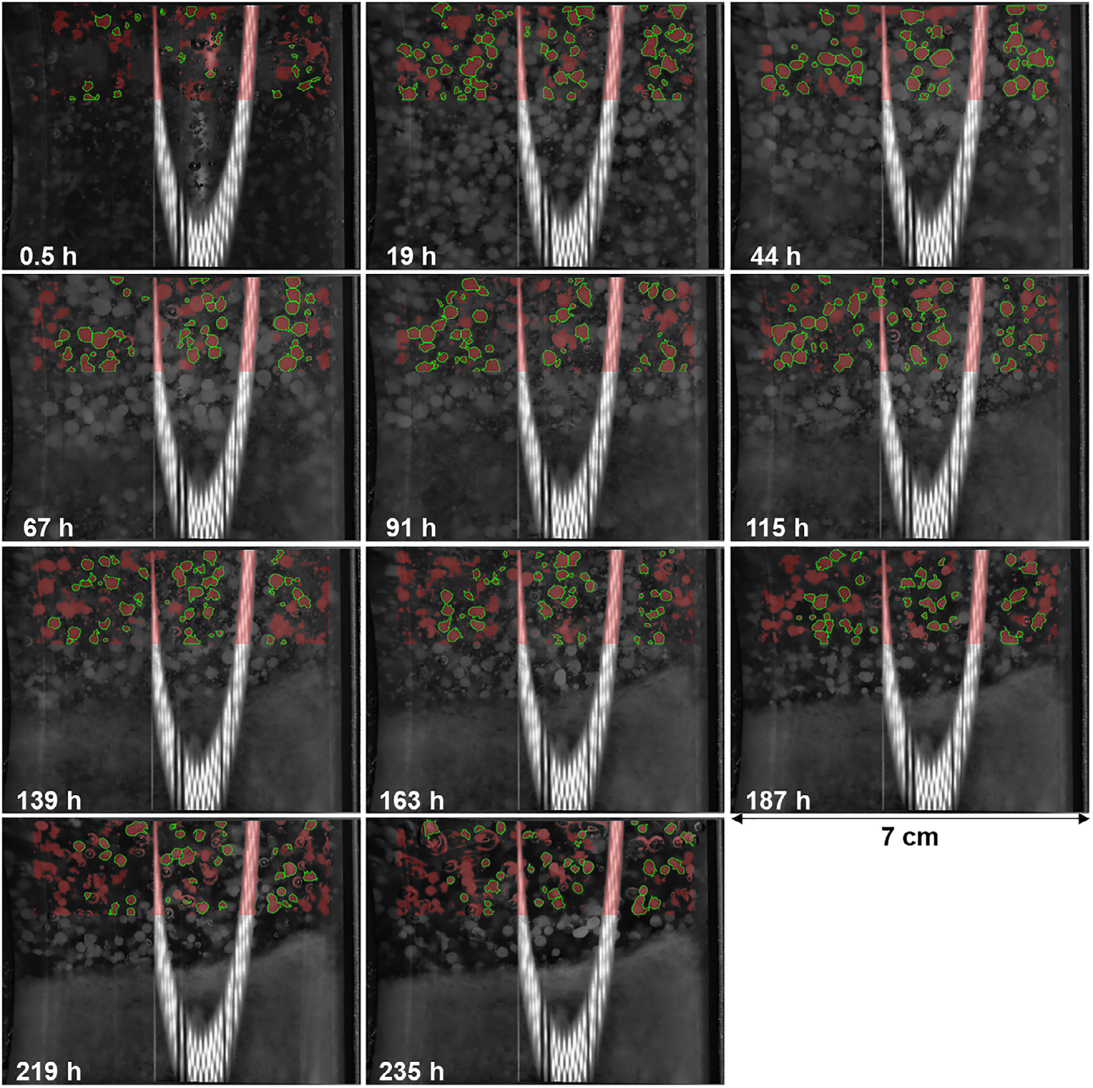
Object detection illustrated with control visualizations of *A. oryzae* DSM 1863 in 2.5 L STR. Cultivation was performed with *X*_0_ of 7.5 g L^−1^ biomass, 109 g L^−1^ glucose, *V*_0_ of 1.4 L, 400 rpm, 32°C, and 1.4 L_n_ min^−1^ air. pH was maintained with 4 M NaOH and 4 M H_3_PO_4_ at 6.50 ± 0.05. Segmented foreground areas after binarization are colored in red. Valid objects are outlined in green. Imaging timestamps correspond to sampling times. Luminosity was measured above the mycelial attachment.

Figure 9 compares pellet size and object abundance monitoring of *A. oryzae* using in situ imaging and offline analysis. For POA, both methods show nearly identical medians immediately after inoculation. Between the initial measurement and 19 h, both in situ and ex situ POA show an increase. For in situ POA, the rise at the onset of phase II coincided with Zn^2+^ limitation. One explanation for this pattern can be pellet coalescence, potentially caused by Zn^2+^ limitation, reducing the number of discrete structures while simultaneously increasing POA, consistent with earlier reports of mature pellets agglomerating [31]. Although the negative hyphal surface charge may contribute to electrostatic repulsion, nutrient deficiency could alter cell wall properties, reducing this constraint [32, 33]. Moreover, the use of NaOH for pH adjustment introduces Na^+^ ions, increasing the ionic strength of the medium, which may increase electrostatic screening, reduce electrostatic repulsion, and thereby facilitate adhesion [34, 35]. Under altered cultivation conditions, pellets may develop a more open and loosely branched architecture [36], potentially increasing their effective surface area for nutrient uptake and consequently the POA. Since trace elements are known to influence fungal morphology, Zn^2+^ limitation provides a plausible explanation for the observed increase in POA [29, 37].

**FIGURE 9:**
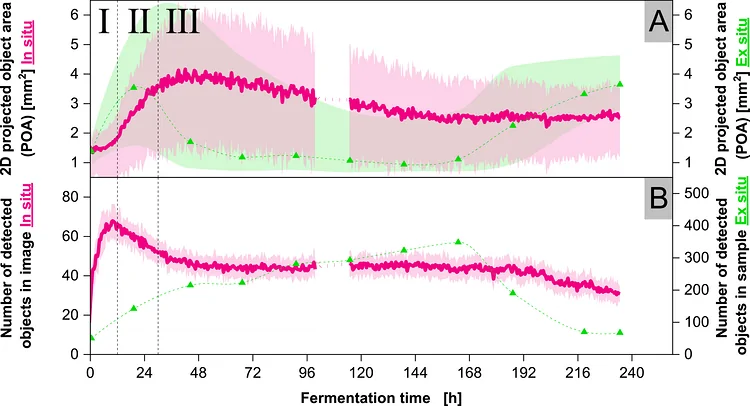
Pellet size and object abundance monitoring of *A. oryzae* DSM 1863 in 2.5 L STR. Cultivation was performed with *X*_0_ of 7.5 g L^−1^ biomass, 109 g L^−1^ glucose, *V*_0_ of 1.4 L, 400 rpm, 32°C, and 1.4 L_n_ min^−1^ air. pH was maintained with 4 M NaOH and 4 M H_3_PO_4_ at 6.50 ± 0.05. Phases I, II, and III are demarcated by dashed lines based on the CTR trajectory shown in Figure 7. Data were obtained by in situ camera imaging (pink) and offline sampling followed by flatbed scanner analysis (green). In panel A, POA distributions are represented by the median and shaded IQR for both methods. For each IAE, the in situ median and IQR were calculated from the POA measurements obtained from 30 images, whereas the ex situ median and IQR were calculated from the POA measurements obtained from each individual sample. In panel B, in situ object abundance is shown as the mean object count across the 30 images of each IAE, with the shaded area representing the standard deviation, whereas ex situ object abundance corresponds to the total number of detected objects in each sample. In situ imaging was unavailable between 101 and 115 h due to a temporary technical issue. Dashed lines and Akima‐spline‐connected deviations are provided for visual guidance. Process data represent a single cultivation.

At the beginning of phase III, in situ POA remains above 3 mm^2^, consistent with the persistence of relatively large pellet structures under nitrogen depletion, whereas ex situ POA declines to 1 mm^2^, likely due to fragmentation and shrinkage during sampling and handling processes that may have been intensified by autolysis under nutrient stress. Toward the end of cultivation, in situ imaging showed largely stable POA, accompanied by a moderate decline in detected object count. This behavior may reflect pellet dynamics under prolonged nutrient limitation and mechanical stress. In contrast, ex situ sampling revealed an increase in median pellet size together with a pronounced reduction in detected object count. The discrepancy between in situ and ex situ trends suggests that the observed increase in ex situ median size is unlikely to reflect true pellet growth within the reactor, but may instead reflect method‐related effects. In particular, sampling and handling may introduce size‐dependent selection, as the pellets persisting at later cultivation stages likely represent a mechanically more robust subpopulation that is less susceptible to fragmentation, resulting in fewer detected objects but larger measured pellet sizes.

### 3.3 Evaluation of the Applied Method

Conventional offline microscopy enables controlled sample preparation and high‐resolution analysis of pellet morphology and hyphal dynamics, including micromorphological features resolved by advanced techniques such as synchrotron‐based x‐ray microtomography [38]. However, such approaches are limited to discrete sampling time points. In dynamic systems, real‐time monitoring and trend detection can provide complementary value despite lower point‐measurement accuracy, as demonstrated in continuous glucose monitoring [39]. Accordingly, the presented in situ imaging approach prioritizes temporal resolution over absolute accuracy to capture dynamic changes in pellet size and object abundance that may be inaccessible to offline methods.

A key limitation of the presented imaging approach is its sensitivity to optical disturbances and morphology‐dependent variations in light scattering, particularly in highly turbid media, in the presence of suspended solids, or when fungal biomass occurs as clumps or dispersed mycelia rather than pellets; these factors may impair image quality or affect luminosity independently of biomass concentration, thereby limiting quantitative analysis. For biomass monitoring in such systems, dielectric spectroscopy represents a robust alternative, as capacitance‐based sensors quantify viable biomass via the polarizable membrane surface and show limited sensitivity to noncellular material [40, 41]. However, these bulk measurements do not directly resolve morphological descriptors and therefore provide only indirect information on pellet size and dynamics. Consequently, imaging‐ and capacitance‐based approaches should be considered complementary.

Our approach enables automated time‐resolved in situ observation of pellet size dynamics and reveals trends that may be distorted by offline sampling. Its comparatively simple hardware and integration requirements make imaging particularly attractive for laboratory‐scale filamentous systems, especially when combined with established biomass measurements to enable complementary assessment of biomass quantity and morphological state. As a potential technical improvement, cross‐polarized illumination using polarizing filters on the light source and camera lens could be employed to reduce specular reflections [42]. Beyond biomass monitoring, imaging supports process understanding and may support process control. Information on pellet size could be integrated into advanced control strategies, enabling dynamic adjustment of parameters such as agitation or aeration to maintain defined pellet structures. In addition, image analysis facilitates detection of process‐specific events, including foaming, color changes associated with secondary metabolite production, biofilm formation, or mycelial attachment [43, 44, 45, 46]. It may also enable early contamination detection, as demonstrated in cell culture [47]. Imaging further allows characterization of gas distribution, such as variations in bubble size, rise velocity, or local gas holdup [48, 49, 50].

## 4 Conclusion

Image‐based analysis represents a promising tool for monitoring biomass and pellet‐size dynamics in microbial cultivation processes. Its ability to operate autonomously in a time‐resolved manner, combined with the potential for real‐time data integration, positions it as a versatile approach for bioprocess observation. Current limitations identified in this study, which should be addressed in future work, include reliance on 2D projections, size‐ and concentration‐dependent deviations likely caused by object overlap and occlusion, and sensitivity to optical disturbances. Despite these limitations, the given insights illustrate how image‐based monitoring can uncover unexpected pellet‐size trends that enable formulation of mechanistic hypotheses, which may not be accessible through conventional biomass measurements alone, all achievable with a simple setup using a standard industrial camera and an ordinary ring light. In future, this method could also be used to enable online control of morphology through adapted control strategies, such as stirring at an altered intensity.

## Author Contributions

**Lukas Hartmann**: conceptualization, methodology, software, visualization, writing – original draft, writing – review and editing. **Tarek Wieland**: conceptualization, methodology, software, data curation, formal analysis, investigation, visualization, writing – review and editing. **Linus Witucki**: conceptualization, methodology, software, writing – review and editing. **Mike Barth**: conceptualization, supervision, project administration, writing – review and editing. **Dirk Holtmann**: conceptualization, supervision, project administration, resources, writing – review and editing.

## Declaration of Generative AI and AI‐Assisted Technologies in the Writing Process

The authors used ChatGPT (OpenAI, San Francisco, California, USA) to support editorial improvements in spelling, grammar, tone, readability, and overall language quality, and to assist in code generation.

## Funding

The authors received no external funding.

## Conflicts of Interest

The authors declare no conflicts of interest.

## Supporting information




**Supporting File**: biot70314‐sup‐0001‐SuppMat.pdf.

## Data Availability

Data are available from the corresponding author upon reasonable request.
