# Supplementary material for: In Situ Imaging for Monitoring Biomass Content and Pellet Size in Submerged Filamentous Stirred‐Tank Cultivation
Source: Biotechnol J. 2026 Sep 28;21(10):e70314. doi: 10.1002/biot.70314 (PMC13620445; doi:10.1002/biot.70314)
Supplement: Supplementary file 1 [file BIOT-21-e70314-s001.pdf]

In Situ Imaging for Monitoring Biomass Content and Pellet Size in Submerged Filamentous Stirred-Tank Cultivation

Lukas Hartmann[+], Tarek Wieland[+], Linus Witucki, Mike Barth, Dirk Holtmann*

[+]The authors contributed equally and share first authorship.

*Corresponding author

Supplementary Material – MATLAB Source Code

The complete MATLAB source code associated with this study is included in this PDF as the embedded attachment MATLAB_source_code.7z. To access the code, please download the PDF and open the attachments panel, indicated by the pin needle icon, in a compatible PDF reader.
